# High yield expression of an AHL-lactonase from *Bacillus *sp. B546 in *Pichia pastoris *and its application to reduce *Aeromonas hydrophila *mortality in aquaculture

**DOI:** 10.1186/1475-2859-9-39

**Published:** 2010-05-21

**Authors:** Ruidong Chen, Zhigang Zhou, Yanan Cao, Yingguo Bai, Bin Yao

**Affiliations:** 1 Key Laboratory for Feed Biotechnology of the Ministry of Agriculture, Feed Research Institute, Chinese Academy of Agricultural Sciences, Beijing 100081, PR China

## Abstract

**Background:**

*Aeromonas hydrophila *is a serious pathogen and can cause hemorrhagic septicemia in fish. To control this disease, antibiotics and chemicals are widely used which can consequently result in "superbugs" and chemical accumulation in the food chain. Though vaccine against *A. hydrophila *is available, its use is limited due to multiple serotypes of this pathogen and problems of safety and efficacy. Another problem with vaccination is the ability to apply it to small fish especially in high numbers. In this study, we tried a new way to attenuate the *A. hydrophila *infection by using a quorum quenching strategy with a recombinant AHL-lactonase expressed in *Pichia pastoris*.

**Results:**

The AHL-lactonase (AiiA_B546_) from *Bacillus *sp. B546 was produced extracellularly in *P. pastoris *with a yield of 3,558.4 ± 81.3 U/mL in a 3.7-L fermenter when using 3-oxo-C8-HSL as the substrate. After purification with a HiTrap Q Sepharose column, the recombinant homogenous protein showed a band of 33.6 kDa on SDS-PAGE, higher than the calculated molecular mass (28.14 kDa). Deglycosylation of AiiA_B546 _with Endo H confirmed the occurrence of *N*-glycosylation. The purified recombinant AiiA_B546 _showed optimal activity at pH 8.0 and 20°C, exhibited excellent stability at pH 8.0-12.0 and thermal stability at 70°C, was firstly confirmed to be significantly protease-resistant, and had wide substrate specificity. In application test, when co-injected with A. *hydrophila *in common carp, recombinant AiiA_B546 _decreased the mortality rate and delayed the mortality time of fish.

**Conclusions:**

Our results not only indicate the possibility of mass-production of AHL-lactonase at low cost, but also open up a promising foreground of application of AHL-lactonase in fish to control *A. hydrophila *disease by regulating its virulence. To our knowledge, this is the first report on heterologous expression of AHL-lactonase in *P. pastoris *and attenuating *A. hydrophila *virulence by co-injection with AHL-lactonase.

## Background

*Aeromonas hydrophila *is a Gram-negative rod and behaves as an opportunistic pathogen in both aquatic and host environments [1-3]. It can cause hemorrhagic septicemia, resulting in fin and tail rot and epizootic ulcerative syndrome in juvenile and mature fish or intestinal and wound infection in humans [3-7]. Application of antibiotics and chemical drugs is a conventional method to control this disease, but generally results in the constant emergence of "superbugs" and chemical accumulation in the food chain [8,9]. Consequently, a new method is required to prevent such a fish disease [8,10]. Several studies have reported that vaccines against *A. hydrophila *infections may provide protection for farmed fish; however, no vaccines are commercially available due to multiple serotypes of this pathogen and problems of safety and efficacy [11,12].

The pathogenicity of *A. hydrophila *depends on the production of potential virulence factors, such as exoproteases and exotoxin [13]. Production of exoproteases is under the control of quorum sensing [1,6]. The findings that *A. hydrophila *harbors the AhyI/AhyR quorum-sensing system, utilizes AHL-dependent quorum-sensing to regulate the expression of virulent genes, and mediates the process of microbial infection and colonization in the host provide a potentially promising strategy to control *A. hydrophila*--quorum-quenching [1,6,7,10,13]. Quorum-quenching mechanism has been identified in many prokaryotic and eukaryotic organisms [10]. It can regulate microbial activities of host by interfering with bacterial quorum sensing [14,15]. Many Gram-negative bacteria produce, secrete, and respond to small diffusible *N*-acyl-homoserine lactone (AHL) signals to communicate with each other and determine group behaviors; for example, bacteria can sense their population density by the concentration of signal molecules and release toxins synchronously for disease outbreak [16,17]. Except for quorum-sensing inhibitors such as furanones and pyrrinones, degradation of quorum-sensing signals by quorum-quenching enzymes is another promising way [17-19]. Quorum-quenching enzymes include AHL-lactonase, AHL-acylase and paraoxonases (PONs) [20-23]. AHL-lactonase, belonging to the metallohydrolase superfamily, catalyzes the hydrolysis of homoserine lactone ring of AHL signals [24,25] and is widely conserved in a range of bacterial species [26]. Unlike AHL-acylase and PONs, which have variable substrate spectra, AHL-lactonase shows distinct substrate specificity and only efficiently hydrolyses AHL signals [20,21,24].

The first AHL-degrading enzyme coding-gene (*aiiA*) was cloned from *Bacillus *sp. 240B1 and expressed in the plant pathogen *Erwinia carotovora *to attenuate *E. carotovora *pathogenicity by reducing AHL accumulation [23]. To date, AHL-lactonases from *Bacillus *spp. have been successfully obtained and expressed in several bacteria (*i.e. *plant pathogen *E. carotovora*, human pathogen *Pseudomonas fluorescens*, insecticide *Bacillus thuringiensis *and recombinant expression strain *Escherichia coli*) and plants (*i.e. *potato and tobacco) to quench quorum sensing of pathogens [27-31]. But little is known about the expression of AiiA in *Pichia pastoris*--a high-yield expression system [32-35]--and the use of AiiA in the form of enzyme preparation to quench quorum sensing of pathogens. Here, we cloned a gene from *Bacillus *sp. B546 encoding an AHL-lactonase and expressed the gene in *P. pastoris *to achieve high-yield production. *A. hydrophila *ATCC 7966, a type strain with the whole genome sequenced [7], is often related with hemorrhagic septicemia in cold-blooded animals including fish, reptiles, and amphibians [36] and was used to infect common carp (*Cyprinus carpio carpio*)--a popular pet fish--by co-injection with the recombinant AHL-lactonase. The attenuating effect of AHL-lactonase on the occurrence of hemorrhagic septicemia in fish was evaluated.

## Results

### Gene cloning and sequence analysis

Using the primers BT1 and BT2 designed specific for AHL-lactonases of *Bacillus *spp., the full-length 753-bp AHL-lactonase gene, *aiiA*_B546_, was cloned from *Bacillus *sp. B546. *aiiA*_B546 _encoded a 250-amino acid polypeptide with a calculated molecular mass of 28.14 kDa and a *pI *of 4.64. No signal peptide was predicted in the deduced amino acid sequence of *aiiA*_B546 _based on SignalP 3.0 analysis. One potential *N*-glycosylation site (Asn-Ser-Thr) was identified at the N terminus by NetNGlyc 1.0 Server. AiiA_B546 _exhibited the maximum amino acid sequence identity (98%) to the AHL-lactonase from *B. thuringiensis *followed by that of *Bacillus *sp. 240B1 (90%).

### Expression and fermentation of recombinant AiiA_B546 _**in ***P. pastoris*

**Figure 1:**
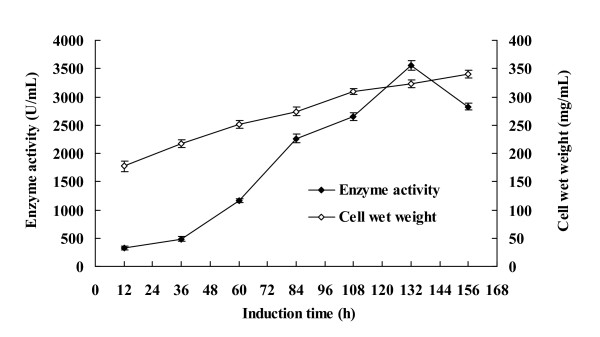
**Accumulation of cell wet weight and enzymatic activity with methanol induction over time in a 3.7-L fermenter**.

The AHL-lactonase gene *aiiA*_B546 _was transformed into *P. pastoris *GS115 competent cells with pPIC9 vector. Positive transformants were screened using well-diffusion assays, and the transformant with the highest AHL-lactonase activity was used for fermentation in the shake flask and 3.7-L fermenter. In the shake-flask level, AHL-lactonase activity was up to 27.1 ± 3.2 U/mL after methanol induction at 25°C for 72 h. In the fermenter, the expression level of recombinant AiiA_B546 _gradually increased with the induction time, and reached 3,558.4 ± 81.3 U/mL after 132 h induction (Figure 1).

### Purification of recombinant AiiA_B546 _in shake-flasks

**Figure 2:**
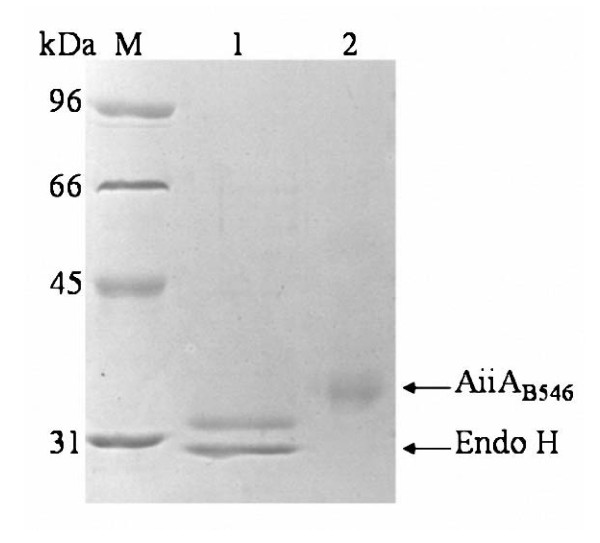
**SDS-PAGE analysis of the purified and deglycosylated AiiA_B546_**. Lanes: M, protein molecular mass standards; 1, the deglycosylated AiiA_B546 _with Endo H; 2, the purified recombinant AiiA_B546_.

Recombinant AiiA_B546 _was purified to electrophoretic homogeneity by ammonium sulfate precipitation and anion exchange chromatography (Figure 2). The molecular weight of the purified AiiA_B546 _was 33.6 kDa based on SDS-PAGE analysis, which was higher than the predicted value (28.14 kDa). The protein concentration and AHL-lactonase activity of the purified recombinant AiiA_B546 _was 0.006 mg/mL and 17.97 ± 2.7 U/mL, respectively. The specific activity of the purified recombinant AiiA_B546 _was 2,995.3 ± 61.7 U/mg with 3-oxo-C8-HSL as the substrate.

### Deglycosylation and LC-ESI-MS/MS analysis

After treatment with Endo H, the deglycosylated AiiA_B546 _migrated as a single band of approximately 31.5 kDa on SDS-PAGE (Figure 2), still higher than the predicted value. To confirm the identity of the purified protein, five interval peptides--KLYFVPAGR, MTEEDRIVNILKR, ENFEDEVPFAGVDSELALSSIKR, KENPIVFFGHDIEQEK and AEYETAQHSEEYLK--were obtained from LC-ESI-MS/MS analysis and shared 100% identity with the deduced amino acid sequence of *aiiA*_B546_.

### Enzyme characterization

**Figure 3:**
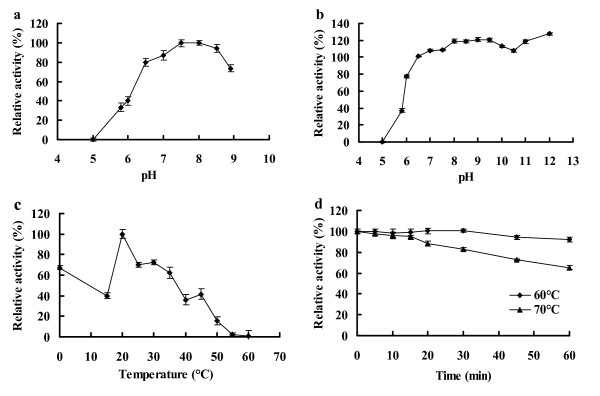
**Enzyme characterization of purified recombinant AiiA_B546_**. a: Effect of pH on the AHL-lactonase activity of AiiA_B546_. b: pH stability assay. After pre-incubating the enzyme at 25°C for 1 h in buffers of pH 5.0-12.0, the residual activity was measured in PBS buffer (pH 8.0) at 25°C. c: Effect of temperature on AHL-lactonase activity. d: Thermostability of recombinant AiiA_B546_. The enzyme was pre-incubated at 60 and 70°C in PBS buffer (pH 8.0), and aliquots were removed at specific time points for the measurement of residual activity at 25°C.

Purified recombinant AiiA_B546 _had the optimum pH of 8.0, and retained more than 73% of the maximum activity at pH 6.5-8.9 (Figure 3a). Recombinant AiiA_B546 _was stable at pH 6.0-12.0, retaining more than 70% activity after pre-incubation at 37°C for 1 h (Figure 3b). The optimum temperature of recombinant AiiA_B546 _was 20°C; at 0 and 20-35°C, the enzyme maintained more than 60% of the highest activity (Figure 3c). Recombinant AiiA_B546 _exhibited thermal stability at 70°C, retaining more than 80% of the initial activity after pre-incubation at 70°C for 30 min (Figure 3d). After storage at 0°C for 3 months, the enzyme still maintained 98.4% of the original activity.

**Figure 4:**
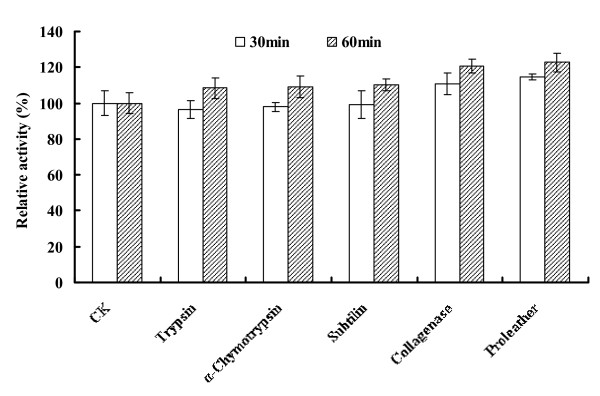
**Effect of proteases on AHL-lactonase activity**. The residual AHL-lactonase activity was determined after treatment with proteases at a ratio of 1:10 (w/w; protease/AiiA_B546_) at 37°C for 30 or 60 min. CK indicates no proteases added.

The purified recombinant AiiA_B546 _was protease-resistant (Figure 4). After incubation with trypsin, subtilisin A, collagenase and proleather at 37°C for 30 or 60 min, the enzyme maintained or enhanced its enzymatic activity

The effect of different metal ions or chemical reagents on the activity of recombinant AiiA_B546 _is displayed in Table 1. A number of metal ions, including Na^+^, K^+^, Ca^2+^, Fe^3+^, Mn^2+^, exhibited positive effects on the enzyme activity at 1 and 10 mM, respectively. AHL-lactonase activity was enhanced by Mg^2+^, Zn^2+ ^and EDTA at 10 mM but reduced at 1 mM, Li^+^, Pb^2+ ^and *β*-mercaptoethanol promoted the enzyme activity at 1 mM but reduced at 10 mM. AHL-lactonase activity was significantly or completely inhibited by Cu^2+^, Cr^3+^, SDS, Hg^2+ ^and Ag^+ ^at both concentrations tested.

**Table 1:** Effects of metal ions and chemicals on the purified recombinant AiiA_B546 _activity

Chemicals	**Relativity activity (%)**^**a**^		Chemicals	**Relativity activity (%)**^**a**^	
	**1 mM**	**10 mM**		**1 mM**	**10 mM**

None	100	100	Mg^2+^	64.8 ± 5.2*	108.4 ± 2.5*
Na^+^	109.5 ± 5.2*	104.6 ± 2.4*	Fe^3+^	104.8 ± 2.6*	103.3 ± 1.7*
K^+^	104.5 ± 2.9*	109.3 ± 6.1*	Zn^2+^	89.3 ± 4.2*	109.5 ± 5.9*
Ca^2+^	106.5 ± 3.7*	117.5 ± 1.2*	Mn^2+^	107.6 ± 4.9*	108.4 ± 5.8*
Li^+^	115.4 ± 3.9*	65.8 ± 5.4*	Ni^2+^	103.4 ± 4.4	102.6 ± 3.5
Co^2+^	118.5 ± 7.1*	103.8 ± 4.3	Pb^2+^	108.6 ± 3.9*	87.5 ± 3.6*
Cu^2+^	68.5 ± 7.2*	58.8 ± 5.4*	EDTA	66.5 ± 2.5*	103.6 ± 3.3*
Cr^3+^	29.1 ± 4.2*	49.6 ± 3.8*	SDS	73.2 ± 4.2*	0.0 ± 5.2*
Ag^+^	0.0 ± 5.2*	0.0 ± 3.7*	β-Mercaptoethanol	112.2 ± 3.8	92.0 ± 5.1*
Hg^2+^	0.0 ± 3.9*	0.0 ± 4.9*			

^a ^Values represent the means ± SD (n = 3) relative to the untreated control samples, and those marked with "*" are significantly different (one-way ANOVA; *P *< 0.05) from that of control without addition of chemicals.

Recombinant AiiA_B546 _exhibited catalytic activities towards all of the tested AHLs, including C10-HSL (211,880 U/mL), C12-HSL (84,450 U/mL), C6-HSL (19,030 U/mL), 3-oxo-C6-HSL (6,450 U/mL), 3-oxo-C8-HSL (27.1 U/mL), and C8-HSL (3.32 U/mL).

### Co-injection of recombinant AiiA_B546 _and A. *hydrophila *in common carp

**Figure 5:**
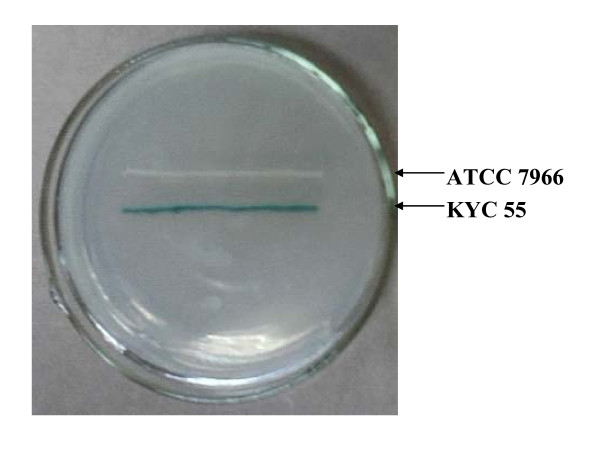
**AHL production in *A. hydrophila *ATCC 7966**. Screening of AHL production in *A. hydrophila *ATCC 7966 by reporter strain *A. tumefaciens *KYC55.

**Figure 6:**
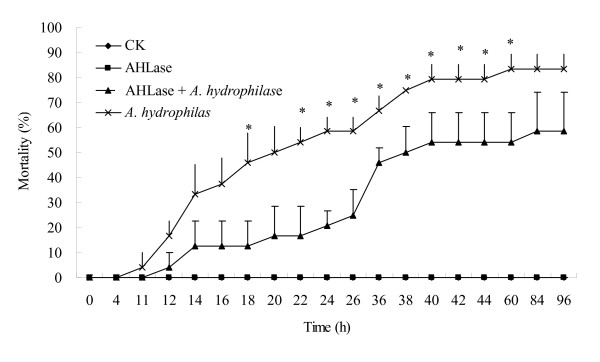
**Average cumulative mortality rate in common carp of four treatments within 4 days**. Average cumulative mortality rate in common carp inoculated with sterile PBS buffer (CK), AiiA_B546 _(0.4 U), *A. hydrophila *(10^8 ^cfu) or AiiA_B546 _(0.4 U) + *A. hydrophila *(10^8 ^cfu). Each point or bar represents the mean of triplicate plus standard deviation, and those marked with "*" means significant difference (one-way ANOVA; *P *< 0.05) among the groups.

*Aeromonas hydrophila *ATCC 7966 was detected AHLs producing positive by reporter strain *Agrobacterium tumefaciens *KYC 55 (Figure 5). The LD_50 _of *A. hydrophila *to common carp was estimated at day 4 after intraperitoneal injection with 10^8 ^cfu. When intraperitoneally injected with PBS buffer or AiiA_B546 _only for 4 days, no mortalities or pathogenic symptoms were observed. When intraperitoneally injected with AiiA_B546 _+ *A. hydrophila*, the accumulated mortality at day 4 was 54.17 ± 11.79%, significantly lower than the average mortality rate (79.17 ± 5.89%) of the fish injected with 10^8 ^cfu of *A. hydrophila *(*P *< 0.05) (Figure 6). Co-injection of AiiA_B546 _and *A. hydrophila *decreased the mortality rate of common carp by nearly 25%. The LT_50 _of the fish injected with *A. hydrophila *was about 20 h, which was delayed to 38 h by co-injection of AiiA_B546 _and *A. hydrophila*.

## Discussion

AHLs-encoding genes (*aiiA*) have been expressed in several pathogens (*i.e. E.carotovora *and *P. aeruginosa*), plants (*i.e. *potato and tobacoo), *B. thuringiensis *and *E. coli *[23,27-31]. Heterogenous expression of *aiiA *in pathogenic bacteria decreased the concentrations of auto-inducers, reduced the expression of several virulence factors (*i.e. *elastase and pyocyanin in *P. aeruginosa*) and attenuated the virulence of pathogens [23,28-30]. Expression of *aiiA *in the insecticide *B. thuringiensis *conferred the strain with a strong biocontrol capacity against AHL-dependent pathogen *E. carotovora *when co-inoculated with the pathogen [37]. *aiiA *was also expressed in *E. coli *and purified to study the specificity of the enzyme [17,24]. The recombinant *E. coli *was able to attenuate the pathogenicity of *E. carotovora *when co-inoculated together [17]. All of these results indicate that *aiiA *encoded a protein against bacterial pathogens by hampering their AHL-dependent quorum sense system. However, these transgenic strains have to be co-inoculated with AHL-dependent pathogens for disease control and consequently influence ecological security and the stable expression of *aiiA *in transgenic strains [31,38]. Considering the achievements and shortcomings of previous studies, we proposed two major innovations in this study. One is that we successfully overexpressed *aiiA *in *P. pastoris *for high-yield fermentation. To our knowledge, this is the first heterologous expression of *aiiA *in *P. pastoris*. The other is that we applied AHL-lactonase directly instead of AHL-lactonase-producing strains to control AHL-dependent pathogens of animals. Co-injection of AiiA_B546 _with *A. hydrophila *in fish successfully attenuated the *A. hydrophila *infection. As far as we know, this is the first study to inject AHL-lactonase to control A. *hydrophila *infection in fish.

Expression of *aiiA*_B546 _in *P. pastoris *enhanced the production of AiiA_B546 _to 3,558.4 ± 81.3 U/mL, which was significantly higher than that expressed in *E. coli *(0.24 U/mL, data of our lab work not published). In addition to high expression level, recombinant AiiA_B546 _expressed in *P. pastoris *had more favorable properties for degrading AHLs *in vivo*. Firstly, recombinant AiiA_B546 _had broad substrate specificity, showing catalytic activity against C4-HSL, C6-HSL, C8-HSL, C10-HSL, C12-HSL, C14-HSL, 3-oxo-C6-HSL and 3-oxo-C8-HSL. Secondly, it showed preferable activity at the temperature range of 20-25°C and neutral and weak alkaline pHs, which are similar to the physicochemical conditions of the carp and their aquatic environment. Thirdly, recombinant AiiA_B546 _expressed in *P. pastoris *was remarkably stable up to 70°C, significantly better than that expressed in *E. coli *(retained 21.3% activity after incubation at 37°C for 30 min, data of our lab work not published). This difference could be ascribed to the post-translation modification of recombinant AiiA_B546 _expressed in *P. pastoris *[39-41]. Deglycosylation with Endo H (Figure 2) confirmed the occurrence of *N*-glycosylation, but didn't account for the complete weight difference. Except for *N*-glycosylation, the recombinant AiiA_B546 _expressed in *P. pastoris *may have other post-translation modifications, such as *O*-deglycosylation and phosphorylation. In addition to excellent thermodynamic stability, recombinant AiiA_B546 _also had other advantageous properties, such as protease-resistance, resistance to many metal ions, and excellent pH stability over a wide pH range. All these properties indicated the application potential of AiiA_B546 _in aquaculture as a good feed additive [42,43].

Many bacterial pathogens use AHL-dependent quorum sensing to regulate virulence, such as the animal pathogen *A. hydrophila *[1,3,6,13], plant pathogen *E. carotovora *[44] and mammalian pathogen *P. aeruginosa *[30,44]. AHL production in pathogen *A. hydrophila *ATCC 7966 has been screened by reporter strain KYC 55 (Figure 5). This result is consistent with that of the *lux*RI homologs of this strain detected by blot hybridization [45]. It has been reported that virulence factors such as extracellular proteases and α-haemolysin are under the control of AHLs [6,13]. Co-injection of AiiA_B546 _and *A. hydrophila *in common carp decreased the mortality rate and delayed LT_50 _of the fish. The results indicated that AiiA_B546 _probably reduced the AHL accumulation of *A. hydrophila*, which would affect related gene expression and allow the host to build up defense mechanism against *A. hydrophila *infection, and eventually contributed to the decreased mortality rate and delayed LT_50 _of the carp observed in the present study [1,6,15]. After injection with the enzyme, fish displayed no signs of stress or disease and no mortalities were observed, thus indicating that the enzyme is safe for carp applications. We assume that the innate immune system of common carp might include some components like AiiA, because the PONs from human and other mammalian species have high catalytic activities against long-chain AHL signals and might be one of the active components of mammalian innate immune systems [21,22,46,47]. When incubated with *A. hydrophila *only, the fish began to die rapidly at 11 h; co-injection of *A. hydrophila *with AiiA_B546 _delayed time to 26 h. We conjectured that AiiA_B546 _probably disrupted *A. hydrophila *transmission in the fish and prevented the pathogen from overcoming the host defenses by destroying the quorum sensing system of *A. hydrophila*. It has been reported that quorum sensing system was used by pathogens to escape premature detection of host defenses and defeat the host successfully at the appropriate time [17,48,49]. The detailed underlying mechanisms of this process, such as which AHLs are hydrolyzed and which virulence genes are hampered, need further study.

## Conclusions

We firstly expressed an AHL-lactonase gene in *P. pastoris *and achieved high-yield fermentation of AHL-lactonase at low cost. The recombinant AHL-lactonase had preferable properties for practical application in aquaculture, such as favorable optimal pH and temperature, excellent pH and temperature stability, high specific activity, good protease-resistance and efficient hydrolysis of AHL signals. We tried a novel application form of AHL-lactonase and successfully controlled *A. hydrophila *infection in carp by using the quorum quenching strategy. Co-injection of AHL-lactonase with *A. hydrophila *decreased the mortality rate and delayed the LT_50_, indicating that the enzyme played an important role in attenuating *A. hydrophila *infection in fish and suggesting a promising way to control outbreaks of *A. hydrophila *disease in aquaculture. This recombinant enzyme with excellent pH and temperature stability and protease-resistance has potential application by feeding in the aquaculture. It is not clear if the same benefit will be observed, and further research about this enzyme in application will be carried out to make it more rigorously and clearly. Direct application of AHL-lactonase by either injection or feeding to control *A. hydrophila *infection might be an effective alternative of antibiotics to avoid the emergence of antibiotic-resistant strains.

## Materials and methods

### Strains and culture conditions

*Bacillus *sp. B546 was isolated from the mud of a fish pond at Wuqing, Tianjin, China using minimal medium [50] containing 3-oxo-C6-HSL as the sole carbon source at 30°C for 6 days. Strain B546 was identified by comparison of its 16S rDNA sequence with known sequences in GenBank and preserved in the China General Microbiological Culture Collection (Beijing, China) under the registered number of CGMCC 3228.

Host strain *P. pastoris *GS115 was purchased from Invitrogen (USA). Minimal methanol medium, minimal dextrose medium, buffered glycerol-complex medium, buffered methanol-complex medium, fermentation basal salts medium, and PTM1 trace salts were prepared as described in the manual of the *Pichia *Expression Kit (Invitrogen).

*Agrobacterium tumefaciens *KYC 55 (pJZ372) (pJZ384) (pJZ410) [51] was used as reporter strain for AHL-degrading activity bioassay. The strain was cultivated at 28°C and 200 rpm for 12 h in LB medium containing 100 mg/mL spectinomycin, 100 mg/mL gentamicin, and 5 mg/mL tetracycline.

*Aeromonas hydrophila *ATCC 7966 was grown in Mueller-Hinton agar (Oxoid; Canada) containing 5% sheep erythrocytes at 30°C [52].

### Plasmids and reagents

The pGEM-T Easy vector (Promega, USA) was used for gene cloning. Plasmid pPIC9 (Invitrogen) was used as expression vector. The DNA purification kit, restriction endonucleases and *T4 *DNA ligase were purchased from TaKaRa (Japan). Trypsin, α-chymotrypsin, subtilisin A, collagenase and proleather were all purchased from Sigma (USA). C4-HSL, C6-HSL, 3-oxo-C6-HSL, C8-HSL, 3-oxo-C8-HSL, C10-HSL, C12-HSL, and C14-HSL were products of Sigma and used as substrates of AHL-lactonase. Other chemicals were of chemical grade and commercially available (Tiangen & GreenFortune, China).

### Cloning and sequencing of the AHL lactonase gene *aiiA*_B546_

The genomic DNA of *Bacillus *sp. B546 was extracted by Bacterial genome extraction kit (Tiangen, China) following the manufacturer's instructions and used as the template for the PCR amplification. Based on the conserved amino acid sequences of AHL lactonases from *Bacillus *spp. and known information [23], a specific primer set was designed as follows: BT1 (5'-GCG**GAATTC**ATGACAGTAAAGAAGCTTTATTTCG-3') and BT2 (5'-ATA**GCGGCCGC**CTATATATACTCTGGGAACAC-3') (the *EcoR*I and *Not*I restriction sites are in blod). The PCR conditions included denaturation at 94°C for 5 min; 30 cycles of 94°C, 30 s, 58°C, 30 s, and 72°C, 1 min; with a final extension at 72°C for 10 min. The PCR product (about 750 bp) was purified and ligated into the pGEM-T Easy vector for sequencing and BLAST analysis.

### Sequence analysis

The nucleotide sequence was analyzed by the Vector NTI Suite10 software. SignalP 3.0 server was used to predict the signal peptide in the deduced amino acid sequence http://www.cbs.dtu.dk/services/SignalP/. The DNA and protein sequences were aligned with known sequences by the blastn and blastp programs http://www.ncbi.nlm.nih.gov/BLAST/, respectively. Glycosylation prediction was performed by NetOGlyc 3.1 and NetNGlyc 1.0 (http://www.cbs.dtu.dk/services/NetOGlyc, NetNGlyc).

### Expression of *aiiA*_B546 _in *P. pastoris*

The recombinant pGEM-T Easy vector harboring *aiiA*_B546 _was digested by *EcoR*I and *Not*I, and cloned into the pPIC9 vector to construct the recombinant plasmid pPIC9-*aiiA*_B546_. The recombinant plasmid was linearized by digestion with *Bgl*II and then transformed into *P. pastoris *GS115 by electroporation at 1.5 kV in a 0.2-cm cuvette. Then, 800 μL ice-cold sorbitol solution was immediately added to the cuvette, and the mixture was incubated at 30°C for 3 days.

Positive transformants were further grown on minimal methanol and minimal dextrose plates at 30°C for 2 or 3 days. The selected transformants were inoculated into 3 mL buffered glycerol-complex medium and cultured at 30°C for 48 h with gentle shaking at 200 rpm. The cells were collected by centrifugation at 5,300 × *g *for 5 min at 4°C and suspended in 1 mL buffered methanol-complex medium. The cells were then cultured at 30°C and 200 rpm for 48 h.

The culture supernatants were subjected to AHL-lactonase activity bioassay to select the clone with highest enzyme activity.

### AHL-lactonase activity bioassay

Reporter strain *A. tumefaciens *KYC 55 was used to evaluate AHL-lactonase activity by using well-diffusion assays. Agar plates for bioassay were prepared by mixing 3 mL of the culture of *A. tumefaciens *KYC 55 and 20 mL LB agar medium (1.2% agar) at 45°C and immediately pouring into a 90-mm petri dish at room temperature. Known volumes of 3-oxo-C8-HSL were added to the wells of 5-mm diameter punched in the middle of the agar plates and incubated at 28°C for 12 h. Sixty-microliter of 50 μg/mL X-gal was spread surrounding the wells for induction. The diameter of induced zones was measured, and the relationship between the amounts of 3-oxo-C8-HSL (Y, nM) and the square of radius (X, mm^2^) is: lnY = 1.381X-10.528 (r^2 ^= 0.9965). The reaction system (200 μL) containing purified recombinant AiiA_B546 _(10 μL) and 24 nM 3-oxo-C8-HSL in 50 mM phosphate buffer (pH 8.0,) was incubated in a water bath at 25°C for 45 min and terminated by addition of 10% SDS to a final concentration of 2%. The reaction mixture was pipetted into agar plate wells to determine the amount of residual 3-oxo-C8-HSL based on the established formula. The reaction system without AiiA_B546 _served as control. One unit (U) of AHL-lactonase activity was defined as the amount of enzyme that hydrolyzed 1 nM of 3-oxo-C8-HSL per minute under the assay conditions.

### Expression and purification of recombinant AiiA_B546 _in shake flasks

The clone with highest enzymatic activity was inoculated into 300 mL buffered glycerol-complex medium and cultured at 30°C for 48 h with gentle agitation at 200 rpm. The cells were collected by centrifugation at 5,300 × *g*, 4°C for 5 min and suspended in 100 mL buffered methanol-complex medium for growth at 30°C and 200 rpm for 72 h. The culture supernatant was collected at 10,000 × *g *at 4°C for 10 min and used for further purification.

Recombinant AiiA_B546_, in the culture supernatant was precipitated with 80% ammonium sulfate saturation, followed by centrifugation at 12,000 × *g *for 10 min, re-suspension in 20 mM Tris-HCl (pH 8.0), and dialysis in the same buffer overnight. The crude enzyme was loaded onto a HiTrap Q Sepharose XL 5 mL FPLC column (GE Healthcare, Sweden) equilibrated with Tris-HCl buffer. Protein was eluted by a linear gradient of NaCl (0-1 M) at a flow rate of 3 mL/min. The fractions with AHL-lactonase activity were collected and identified on SDS-12% PAGE. The protein concentration of the purified recombinant AiiA_B546 _was assayed by the Bradford method [53] with bovine serum albumin as standard. Enzyme activity of the purified recombinant AiiA_B546 _was measured by using well-diffusion assays (3-oxo-C8-HSL as the substrate).

### Deglycosylation and LC-ESI-MS/MS analysis

The purified recombinant AiiA_B546 _was deglycosylated by Endo H at 37°C for 1 h following the manufacturer's instructions (New England Biolabs, USA). The deglycosylated enzyme was analyzed by SDS-PAGE. To identify the purified protein, the relevant protein band was excised from the SDS-PAGE gel, digested with trypsin, and analyzed by LC-ESI-MS/MS (Thermo Finnigan, USA). The results of LC-ESI-MS/MS were compared with the deduced amino acid sequence of *aiiA*_B546_.

### Enzyme characterization of purified recombinant AiiA_B546_

To study the enzyme properties of purified recombinant AiiA_B546_, 3-oxo-C8-HSL was used as the substrate, and the reaction was carried out as described above.

The effect of pH on enzyme activity was determined at 25°C in buffers of pH ranging from 5.0 to 9.0. To study the effect of pH on enzyme stability, the purified enzyme was pre-incubated in different buffers of pH ranging from 5.0 to 12.0 at 37°C for 1 h and measured the AHL-lactonase activity under standard conditions. The buffers used were 0.1 M phosphate buffer for pH 5.0-8.0, 0.1 M Tris-HCl buffer for pH 8.0-9.0, and 0.1 M glycine-NaOH buffer for pH 9.0-12.0.

The optimum temperature of purified recombinant AiiA_B546 _was determined at the optimum pH over the temperature range from 0 to 60°C. Thermal stability was determined by measuring the residual enzyme activity under standard conditions after pre-incubation in 0.1 M phosphate buffer (pH 8.0) at 60°C and 70°C for various durations.

The effects of various metal ions and chemical reagents on the enzymatic activity of purified recombinant AiiA_B546 _were examined at 25°C in 0.1 M phosphate buffer (pH 8.0) containing 1 or 10 mM tested chemicals. The remaining enzyme activity was measured under the standard conditions as described above.

To determine resistance to proteolysis, purified recombinant AiiA_B546 _was incubated with either trypsin or α-chymotrypsin in 0.1 M Tris-HCl (pH 7.0), collagenase or subtilisin A in 0.1 M Tris-HCl (pH 7.5), or proleather in 0.1 M glycine-NaOH buffer (pH 10.0) at 37°C for different periods (30 or 60 min) at a ratio of 1:10 (w/w; protease/AiiA_B546_). Protease resistance was determined by measuring the residual enzyme activity after protease treatment.

The substrate specificity of purified recombinant AiiA_B546 _was studied by measuring the enzyme activity against AHLs with different acyl chain length and substitution. The substrates contained C6-HSL, C8-HSL, C10-HSL, C12-HSL, 3-oxo-C6-HSL, and 3-oxo-C8-HSL.

### Fermentation of recombinant AiiA_B546 _in fermenter

Fermentation of the clone with the highest AHL-lactonase activity in shake flask was performed in a 3.7-L fermenter (Bioengineering KLF 2000, Switzerland). The colony was cultured in 40 mL yeast peptone dextrose (YPD) medium at 30°C, 200 rpm for 48 h and then transferred into 200 mL YPD for growth (30°C, 200 rpm) overnight to prepare the fermentation seed. The seed was inoculated into 2-L of basal salt medium containing PTM1 trace salt solution in the 3.7-L fermenter. The temperature was set at 30°C and the pH was maintained at 5.0 with 28% (v/v) NH_3_•H_2_O. The dissolved oxygen concentration was controlled by airflow and agitation.

The fermentation process was in reference of the Pichia Fermentation Process Guidelines (Invitrogen). Until the initial glucose in the fermentation medium was completely exhausted and the dissolved oxygen concentration level was up to 80%, a glucose-fed batch phase was started by a 25% (w/v) glycose feed at the rate of 36 mL/h/L for 4 h. In succession, the mixture containing 8:1 (v/v) 25% glucose/methanol was added at the rate of 9 mL/h/L for about 4 h; during this phase, the cells adapted to grow in methanol. When the dissolved oxygen concentration increased notably, the mixture was replaced by 100% methanol to initiate the methanol fed-batch phase. The final concentration of 100% methanol was about 0.3% (v/v) for 156 h, and the recombinant protein was induced during this phase. In these three phases, the dissolved oxygen concentration was kept above 20%.

During the induction and expression phase, culture samples were collected every day, and enzyme activity in the supernatant and cell wet weight were measured. The protein content in the supernatant was analyzed by SDS-PAGE.

### Application in the control of bacterial disease in aquaculture

*Aermonas hydrophila *ATCC 7966 and *A. tumefaciens *KYC 55 were streaked in parallel on LB plates containing X-gal to screen strains producing AHL. Common carp of 1.3 ± 0.15 g were raised at the density of 8 fish/tank (10 L/tank) in an indoor recirculation aquaculture system with daily aeration by feeding with commercial dry food (Fishmeal 47.0%, soybean meal 24.0%, wheat flour 24.0%, fish oil 2.0%, Ca(H_2_PO_4_)_2 _2.2%, vitamin/mineral premix [54] 0.8%; proximate composition: crude protein 42.0%, crude lipid 7.2%) at a fixed supply of 0.7% of their body weight every day [55]. The water temperature was kept constant at 28 ± 1°C.

Cells of *A. hydrophila *ATCC 7966 grown in Mueller-Hinton agar containing 5% sheep erythrocytes at 220 rpm at 30°C for 12 h were washed with sterile PBS buffer (pH 7.3) three times, and suspended in PBS buffer as injection preparation. Purified recombinant AiiA_B546 _was suspended in PBS buffer (pH 7.3) before use.

To assess the lethal dose 50% (LD_50_) of *A. hydrophila *ATCC 7966, duplicated groups of 8 common carp (1.3 ± 0.15 g) were injected with 50 μL of serial dilutions of *A. hydrophila *ranging from 2.0 × 10^8 ^to 2.0 × 10^10 ^cfu/mL, and control groups were injected with the same volume of PBS (pH7.3). Cumulative mortality was recorded for 4 days.

To determine if recombinant aiiA_B546 _had any influence on bacterial virulence, four treatments of common carp were designed and the fish of different groups injected intraperitoneally with 0.4 U AiiA_B546_, 0.4 U AiiA_B546 _+ *A. hydrophila *of 10^8 ^cfu (AHLase + *A. hydrophila*), *A. hydrophila *of 10^8 ^cfu (*A. hydrophila*) or PBS buffer (control treatment) per fish in the volume of 50 μL when anesthetized by tricaine methanesulfonate (MS-222). Each treatment included triplicate groups and each group contained 8 fish. The system was aerated, and any dead specimens were removed daily for routine bacteriological examination [56]. Cumulative mortality of each treatment was recorded about every two hours for 4 days.

### Nucleotide sequence accession number

The nucleotide sequence for the *N*-acyl homoserine lactonase gene (*aiiA *_B546_) from *Bacillus *sp. B546 has been deposited in the Genbank under accession no. GQ899185.

## Abbreviations

QS: quorum sensing; QQ: quorum quenching; AHLs: *N*-acyl homoserine lactones; AiiA: *N*-Acyl homoserine lactone lactonase; PONs: paraoxonases; YPD: yeast peptone dextrose medium; C4-HSL: *N*-butanoyl-L-homoserine lactone; C6-HSL: *N*-hexanoyl-L-homoserine lactone; 3-oxo-C6-HSL: *N*-(3-oxohexanoyl)-L-homoserine lactone; C8-HSL: *N*-octanoyl-L-homoserine lactone; 3-oxo-C8-HSL: *N*-*β*-oxooctanoyl-L-homoserine lactone; C10-HSL: *N*-decanoyl-L-homoserine lactone; C12-HSL: *N*-dodecanoyl-L-homoserinelactone; C14-HSL: *N*-3-tetradecanoyl-L- homoserine lactone; MS-222: tricaine methanesulfonate; Endo H: endo-β-*N*-acetylglucosaminidase H; LC-ESI-MS/MS: liquid chromatography-electrospray ionization-tandem mass spectrometry; AHLase: *N*-Acyl homoserine lactonase; LD_50_: half lethal concentration; LT_50 _: half lethal time.

## Competing interests

The authors declare that they have no competing interests.

## Authors' contributions

RD performed the experiment and participated in data analysis and writing of the manuscript. ZZ and YC participated in the design of the research and writing of the manuscript. YB performed fermentation of recombinant AiiA_B546 _in fermenter and participated in data analysis. BY participated in the design of the research and editorial supervision of the manuscript. All authors have read and approved the final version of the manuscript.
